# Symptombelastung bei chronischer Nierenerkrankung – nephrologische Einschätzung im Vergleich zur Betroffenenperspektive (SOMA.CK-Studie)

**DOI:** 10.1007/s00108-026-02155-9

**Published:** 2026-07-22

**Authors:** Birte Jessen, Bernd Löwe, Christian Schmidt-Lauber, Tobias B. Huber, Meike Shedden-Mora

**Affiliations:** 1 Institute for Clinical Psychology and Psychotherapy und Department Psychologie, Medical School Hamburg, Am Kaiserkai 1, 20457 Hamburg, Deutschland; 2 Klinik und Poliklinik für Psychosomatische Medizin und Psychotherapie, Universitätsklinikum Hamburg-Eppendorf, Hamburg, Deutschland; 3 III. Medizinische Klinik und Poliklinik, Universitätsklinikum Hamburg-Eppendorf, Hamburg, Deutschland; 4 Hamburg Center for Kidney Health (HCKH), Universitätsklinikum Hamburg-Eppendorf, Hamburg, Deutschland

**Keywords:** Betroffenperspektive, Perspektive von Nephrologinnen und Nephrologen, Chronic Kidney Disease Symptom Burden Index (CKD-SBI), Symptomerfassung, SOMA.CK, Patients’ perspective, Nephrologists’ perspective, Chronic Kidney Disease Symptom Burden Index (CKD-SBI), SOMA.CK

## Abstract

**Hintergrund:**

Menschen mit nichtdialysepflichtiger chronischer Nierenerkrankung („non-dialysis chronic kidney disease“ [ND-CKD]) berichten häufig eine hohe körperliche Symptombelastung. Traditionelle klinische Ansätze postulieren eine hohe Symptombelastung erst in späten Stadien.

**Fragestellung:**

Diese Studie vergleicht die Symptombelastung bei ND-CKD aus ärztlicher Sicht mit der Patientenperspektive.

**Material und Methoden:**

In einer deutschlandweiten Onlinebefragung benannten Nephrologinnen und Nephrologen die drei häufigsten Symptome sowie die durchschnittliche Symptombelastung bei chronischer Nierenerkrankung („chronic kidney disease“ [CKD]) mithilfe einer adaptierten Version des Chronic Kidney Disease Symptom Burden Index (CKD-SBI). Die Ergebnisse wurden mit Selbstauskünften im CKD-SBI von Personen der SOMA.CK-Studie mit ND-CKD verglichen.

**Ergebnisse:**

Einschätzungen zur Symptombelastung von *N* = 108 Nephrologinnen und Nephrologen (durchschnittliche Berufserfahrung: 14,3 Jahre) wurden mit den Angaben von *N* = 222 Personen mit ND-CKD (Durchschnittsalter = 61,22 Jahre, Standardabweichung [SD] = 14,90) verglichen. Nephrologinnen und Nephrologen nannten Fatigue (69,44 %), Ödeme (38,89 %) und arterielle Hypertonie (35,19 %) am häufigsten, während Betroffene Unterbrechung des Nachtschlafs durch Harndrang (74,30 %), Fatigue (61,30 %) und Schlafprobleme (47,30 %) angaben. Nephrologinnen und Nephrologen überschätzten sowohl die Gesamtsymptombelastung als auch die Symptombelastung von 5 Einzelsymptomen signifikant, während sie die von 12 Einzelsymptomen unter- und die von 15 Einzelsymptomen vergleichbar einschätzten.

**Schlussfolgerung:**

Eine Diskrepanz zwischen ärztlich eingeschätzter und patientenberichteter Symptombelastung bei ND-CKD wird deutlich. Die Ergebnisse stützen die Empfehlung, die Betroffenenperspektive in der Symptomerfassung stärker zu berücksichtigen.

**Zusatzmaterial online:**

Zusätzliche Informationen sind in der Online-Version dieses Artikels (https://doi.org/10.1007/s00108-026-02155-9) enthalten.

## Abstract (en)

**Background:**

Individuals with nondialysis chronic kidney disease (ND-CKD) often report high somatic symptom burden, though traditional clinical approaches associate symptom burden only with advanced disease stages.

**Objectives:**

This study compared the symptom burden in ND-CKD as perceived by physicians and patients.

**Materials and methods:**

In a German-wide online survey, nephrologists identified the three most common symptoms and the mean symptom burden in CKD using an adapted version of the Chronic Kidney Disease Symptom Burden Index (CKD-SBI). Their assessments were compared with patients’ self-reports (CKD-SBI) from the SOMA.CK study.

**Results:**

Responses from *N* = 108 nephrologists (average professional experience = 14.3 years) were compared with responses from *N* = 222 individuals with ND-CKD (average age = 61.22 years, standard deviation [SD] = 14.90). Nephrologists most frequently named fatigue (69.44%), oedema (38.89%) and arterial hypertension (35.19%), while patients named nycturia (74.30%), fatigue (61.30%) and sleeping problems (47.30%). Nephrologists significantly overestimated the overall symptom burden and the symptom burden of five single symptoms, while they assessed the severity of 12 symptoms lower and 15 symptoms as comparable.

**Conclusion:**

A discrepancy between the symptom burden reported by physicians and patients with ND-CKD was shown. Thus, giving greater consideration to the patients’ perspectives in symptom assessment is recommended.

## Symptombelastung bei chronischer Nierenerkrankung

Die chronische Nierenerkrankung („chronic kidney disease“ [CKD]) ist gekennzeichnet durch Anomalien der Nierenfunktion oder -struktur, die mindestens 3 Monate andauern. Sie stellt mit einer geschätzten Prävalenz von etwa 10 % ein nationales und globales Gesundheitsproblem dar [10, 20]. Anhaltende körperliche Beschwerden, auch als persistierende somatische Symptome (PSS) bezeichnet [11, 12], kommen in allen Stadien der CKD vor und beeinflussen substanziell die Lebensqualität, Morbidität und Mortalität [22].

Neuere Studien zeigen eine hohe Symptombelastung bereits bei nichtdialysepflichtiger CKD

Während die CKD traditionell bis zum Endstadium als überwiegend asymptomatisch angesehen wurde, zeigen neuere Studien eine hohe Symptombelastung bereits bei nichtdialysepflichtiger CKD („non-dialysis CKD“ [ND-CKD]; [3, 7, 23]). Neben körperlichen Symptomen erleben Personen mit CKD in allen Stadien eine erhöhte Belastung durch psychische Symptome wie depressive Stimmung und Ängstlichkeit [7, 10].

## Diskrepanz in der Symptomerfassung

Die Leitlinien von Kidney Disease Improving Global Outcomes (KDIGO; [10]) sowie die Living Well With Kidney Disease and Effective Symptom Management Consensus Conference 2022 [18] fordern entsprechend ein aktives Symptommanagement [10, 18] und eine interdisziplinäre Behandlung [10, 18, 21], wobei unterschiedliche fachärztliche Expertise, etwa aus Anästhesie oder Diabetologie, sowie psychologische Expertise einbezogen werden sollte. Aktuelle Forschung deutet jedoch auf eine Diskrepanz zwischen wahrgenommener Symptombelastung aus Patientenperspektive und ärztlicher Sicht hin, unter anderem durch unzureichende Dokumentation von Symptomen und mangelnden Einsatz standardisierter Erhebungsinstrumente bei ND-CKD. Außerdem besteht eine Diskrepanz zwischen den Patientenberichten und ärztlichen Auffassungen hinsichtlich der Symptombelastung bei CKD. Vorhergehende Arbeiten berichten, dass nephrologisches Fachpersonal die Symptombelastung von Betroffenen mit CKD häufig unterschätzt [4, 17, 26, 27]. Weisbord et al. [27] fanden heraus, dass von 30 Symptomen 29 hinsichtlich ihres Vorhandenseins und 19 in ihrer Schwere unterschätzt wurden. Zumindest bei Betroffenen mit Hämodialyse schienen Pflegekräfte die Symptombelastung besser einzuschätzen als Ärztinnen und Ärzte [17]. Bisherige Studien untersuchten primär die terminale Niereninsuffizienz, was die Dringlichkeit einer routinemäßigen Symptomerfassung [9, 27] und einer interdisziplinären Zusammenarbeit zur Verbesserung des Symptommanagements auch bei ND-CKD betont [14].

Ziel der vorliegenden Studie ist daher, die Symptomwahrnehmung bei ND-CKD aus Sicht von Nephrologinnen und Nephrologen mit der wahrgenommenen Symptombelastung von Betroffenen zu vergleichen.

## Methoden

### Studiendesign

Diese Fachpersonenumfrage ist Teil der SOMA.CK-Studie [22], die Prädiktoren persistierender somatischer Symptome bei ND-CKD (Stadium 2–4) in einem prospektiven Beobachtungsdesign untersucht. SOMA.CK ist ein Teilprojekt der SOMACROSS-Forschungsgruppe FOR 5211 [11], die Mechanismen von PSS bei verschiedenen Erkrankungen erforscht. Das Studienprotokoll wurde von der Ethikkommission der Landesärztekammer Hamburg genehmigt (Referenznummer 2020-10195-BO-ff) und bei ISRCTN präregistriert (ISRCTN16137374). Die Studie folgt den Strengthening-the-Reporting-of-Observational-Studies-in-Epidemiology(STROBE)-Richtlinien.

### Teilnehmende und Rekrutierungsstrategie

Nephrologinnen und Nephrologen in Deutschland wurden per E‑Mail über die Studie informiert. Zusätzlich erfolgte eine Bewerbung in der Mitgliederinformation der Deutschen Gesellschaft für Nephrologie (DGfN) im August 2023. Die Teilnahme war freiwillig und anonym. Teilnehmende mit ND-CKD wurden über Kooperationskliniken der SOMA.CK-Studie rekrutiert [22]. Von den 364 angesprochenen Personen mit ND-CKD wurden 222 in die SOMA.CK-Studie eingeschlossen. Die Rücklaufquoten zu den Follow-up-Erhebungszeitpunkten lagen bei 93,7 % (Follow-up T1 nach 6 Monaten) und 90,5 % (Follow-up T2 nach 12 Monaten). Drop-out-Gründe vor Einschluss waren unter anderem ein zu hoher Aufwand für die Studie oder Nichterfüllen der Einschlusskriterien sowie Unerreichbarkeit oder Tod zu den Folgezeitpunkten.

### Setting und Studienablauf

Zwischen März und Dezember 2023 konnten Nephrologinnen und Nephrologen an einer 5‑ bis 7‑minütigen anonymen Onlinebefragung in Tivian [24] teilnehmen. Zu Beginn wurden die Studienziele erläutert und die Teilnehmenden um Studieneinwilligung gebeten. Teilnehmende mit ND-CKD wurden von Mai 2022 bis Januar 2025 im Rahmen der Baseline-Erhebung der SOMA.CK-Studie befragt.

### Datenerhebung

Nephrologinnen und Nephrologen wurden um Einschätzung der häufigsten Symptome, der durchschnittlichen Symptombelastung sowie der häufigen Annahmen zur Symptombelastung ihrer Patientinnen und Patienten mit ND-CKD gebeten.

#### Häufigste Symptome.

Nephrologinnen und Nephrologen sollten die drei aus ihrer Sicht häufigsten Symptome bei Personen mit CKD-Stadium 2–4 in offenem Fragenformat benennen.

#### Nierenspezifische Symptombelastung.

Der Chronic Kidney Disease Symptom Burden Index (CKD-SBI) ist ein nierenspezifischer Selbstberichtfragebogen, der *das Vorhandensein sowie die Belastung, Ausprägung und Häufigkeit* von 32 somatischen und psychischen Symptomen auf einer Skala von 0 bis 10 erfasst. Für die vorliegende Analyse wurde ausschließlich die Belastungsskala genutzt. Für die Befragung der Nephrologinnen und Nephrologen wurde der CKD-SBI adaptiert: *„Wie beeinträchtigt sind Ihre Patienten und Patientinnen mit einer chronischen Nierenerkrankung in Stadium 2–4 im Durchschnitt durch folgende Symptome?“*; Antwortoptionen von 0 (*überhaupt nicht beeinträchtigt*) bis 10 (*extrem beeinträchtigt*; [1, 2]). Ein Gesamtscore aus somatischen und psychischen Symptomen wurde berechnet. Einzelsymptome werden getrennt berichtet.

#### Annahmen zur Symptombelastung.

Fünf Aussagen über gängige Vorstellungen zur Symptombelastung bei Personen mit CKD wurden entwickelt. Die Nephrologinnen und Nephrologen konnten diese auf einer 5‑stufigen Skala mit *starker Zustimmung* bis *starker Ablehnung *bewerten (Abb. 2).

#### Soziodemografische Daten.

Soziodemografische Daten des Fachpersonals wie Alter, Geschlecht, Berufserfahrung und die durchschnittliche Arbeitszeit pro Woche mit CKD-Betroffenen wurden erfasst.

#### Routinemäßige Symptomanamnese.

Nephrologinnen und Nephrologen sollten offene Beispielfragen zur Symptomanamnese für typische Konsultationen von Patientinnen und Patienten mit CKD nennen, um das Vorgehen der Symptomerfassung in der nephrologischen Praxis besser zu verstehen.

Patientinnen und Patienten mit ND-CKD wurden im Rahmen der SOMA.CK-Studie zur eigenen nierenspezifischen Symptombelastung mittels CKD-SBI befragt [22]. Zudem wurden soziodemografische und erkrankungsbezogene Daten erfasst: Alter, Geschlecht, Bildungsstand, geschätzte („estimated“) glomeruläre Filtrationsrate (eGFR), berechnet mit der Formel 2021 Chronic Kidney Disease Epidemiology Collaboration (CKD-EPI) Kreatinin; 2009 CKD-EPI Kreatinin ohne ethnische Zugehörigkeit; kreatininbasierte eGFR unter Berücksichtigung von Alter und Geschlecht („sex“) – eGFRcr(AS), neu [8]. Der Bildungsstand wurde aus der Variablen des höchsten Schulabschlusses in drei Kategorien eingeteilt: *gering* (= noch in der Schule, kein Schulabschluss, Sonderschule/Förderschule oder Haupt‑/Volksschulabschluss), *mittel* (= polytechnische Oberschule oder Realschulabschluss), *hoch* (= Fachabitur, Abitur oder abgeschlossenes Studium). Sonstige Schulabschlüsse wurden individuell zugeteilt.

### Datenanalyse

In der Nephrologenbefragung wurden aus Freitextangaben der häufigsten Symptome Häufigkeiten berechnet, indem die Anzahl der Nennungen eines jeweiligen Symptoms ins Verhältnis zur Gesamtstichprobe gesetzt wurde. Quantitative Angaben aus dem CKD-SBI beider Gruppen wurden mittels Summenscore und Konfidenzintervallen analysiert. Die Gesamtsymptombelastung sowie die Symptombelastung der Einzelsymptome wurden mittels t‑Test zwischen den Gruppen verglichen, wobei nichtsignifikante t‑Werte für eine vergleichbare Einschätzung, negative t‑Werte für eine Unterschätzung und positive t‑Werte für eine Überschätzung der Symptombelastung durch Nephrologinnen und Nephrologen stehen. Symptomhäufigkeiten aus Einzelsymptomen des CKD-SBI wurden berechnet und mittels Odds Ratio in logistischen Regressionen mit Angaben von Teilnehmenden der SOMA.CK-Studie verglichen. Symptome mit 100 %iger Nennung wurden wegen potenziell unendlicher Schätzwerte mittels Chi-Quadrat-Test verglichen. Fragen zur routinemäßigen Symptomanamnese wurden in fünf Kategorien eingeteilt:

- Allgemeine Fragen zum Wohlbefinden
- Fragen zur Funktionalität
- Allgemeine symptombezogene Fragen
- Spezifische symptombezogene Fragen
- Sonstige

Statistische Analysen wurden mit SPSS Version 27 durchgeführt.

## Ergebnisse

### Stichprobenbeschreibung

Es nahmen 108 Nephrologinnen und Nephrologen teil. Das Durchschnittsalter betrug 48,37 Jahre (Standardabweichung [SD] = 10,71), 68 (63 %) waren männlich. Die durchschnittliche Berufserfahrung betrug 15,54 Jahre (SD = 9,96). Ein Anteil von 77,8 % hatte mehr als 10 h/Woche Kontakt mit Personen mit ND-CKD, bei 21 (19,4 %) waren es 5–10 h/Woche und bei 3 (2,8 %) 2–5 h/Woche. 100 (92,59 %) waren bereits Fachärztinnen und Fachärzte, 8 (7,41 %) befanden sich noch in der Facharztausbildung, 75 (69,4 %) gaben an, promoviert zu haben, und 17 (15,7 %), habilitiert zu sein.

Zur Baseline der SOMA.CK-Studie nahmen 222 Betroffene mit ND-CKD teil, die sich auf alle CKD-Stadien verteilten (eGFR: *M* = 49,90, SD = 24,33, Spannweite = 12,75–123,53), wobei sich 15 (6,8 %) in Stadium 1, 40 (18,0 %) in Stadium 2, 54 (24,3 %) in Stadium 3a, 53 (23,9 %) in Stadium 3b, 47 (21,2 %) in Stadium 4 und 1 (0,5 %) in Stadium 5 befanden. Bezüglich der Genese der Nierenerkrankung lag bei 23 (10,4 %) ein Diabetes, bei 47 (21,2 %) eine Hypertonie, bei 46 (20,7 %) eine Glomerulonephritis, bei 34 (15,32 %) eine polyzystische Nierenerkrankung und bei 72 (32,4 %) eine andere Erkrankung zugrunde. Das Durchschnittsalter betrug 61,72 Jahre (SD = 14,93); 51 (23,0 %) Personen hatten einen niedrigen, 57 (25,7 %) einen mittleren und 114 (51,4 %) einen hohen Bildungsstand.

### Einschätzung der Top-3-Symptome bei ND-CKD

Nephrologinnen und Nephrologen nannten im Freitextformat *Fatigue* (69,44 %), *Ödeme* (38,89 %) und *arterielle Hypertonie* (35,19 %) als häufigste Symptome, während Betroffene laut CKD-SBI *Unterbrechung des Nachtschlafs durch Harndrang* (74,30 %), *Fatigue* (61,30 %) und *Probleme beim Durchschlafen* (47,30 %) angaben. Alle Freitextantworten sind in Tab. S1 zusammengefasst.

### Vergleich der Symptombelastung

**Abb. 1:**
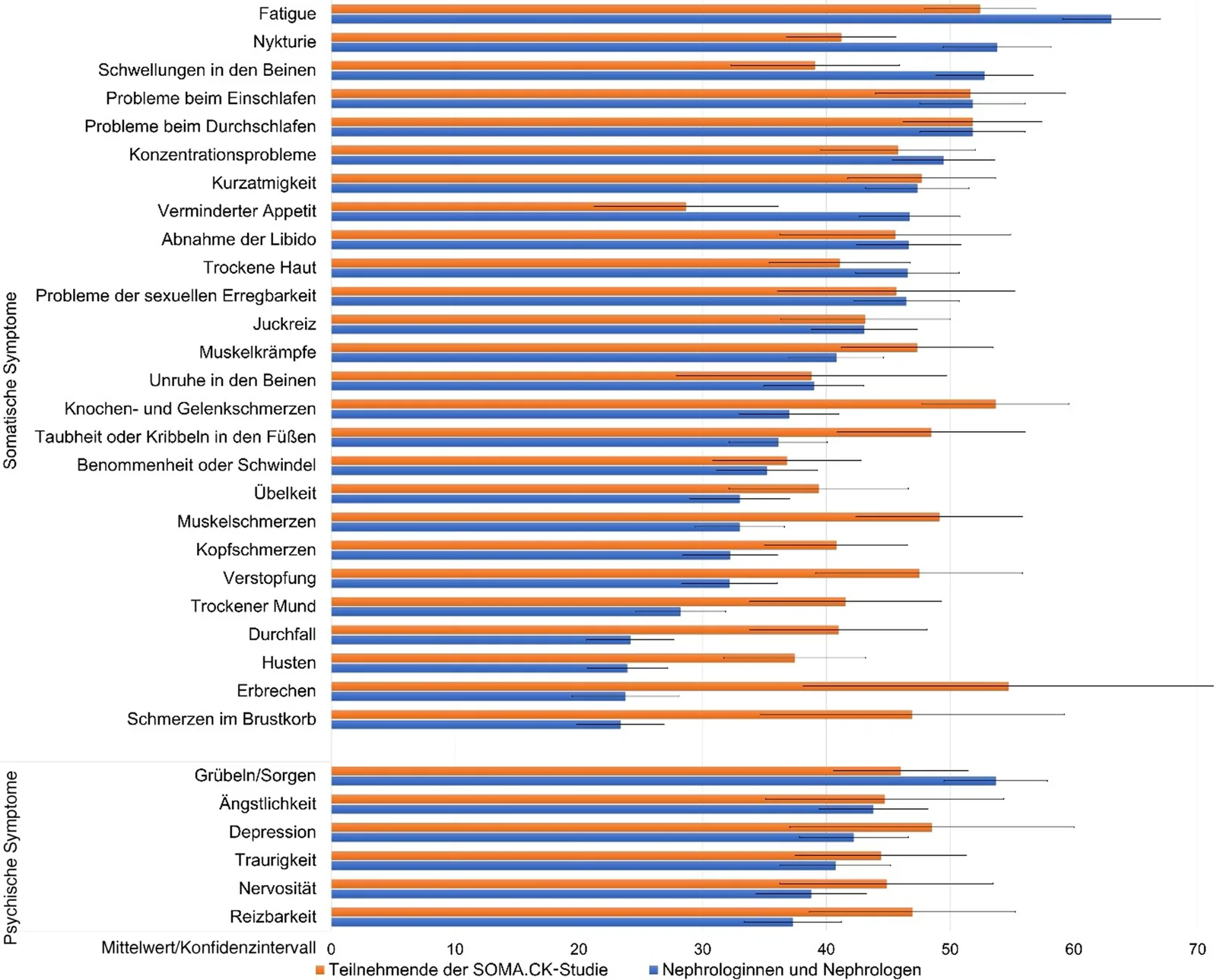
Durchschnittliche Symptombelastung nach Chronic Kidney Disease Symptom Burden Index (CKD-SBI) – Einschätzung durch Nephrologinnen und Nephrologen vs. Personen mit nichtdialysepflichtiger chronischer Nierenerkrankung getrennt nach somatischen und psychischen Symptomen

Nephrologinnen und Nephrologen schätzten sowohl die Anzahl der Symptome (*M* = 30,36, SD = 3,91 vs. *M* = 9,45, SD = 6,10; *t*(304,05) = 37,59; *p* < 0,001) als auch die durchschnittlich wahrgenommene Gesamtsymptombelastung (*M* = 119,31, SD = 47,35 vs. *M* = 42,55, SD = 40,43; *t*(185,10) = 14,48; *p* < 0,001) signifikant höher ein als die Betroffenen. Beim Vergleich der durchschnittlichen Symptombelastung durch Einzelsymptome schätzten Nephrologinnen und Nephrologen die Belastung bei 15 Symptomen (unter anderem Kurzatmigkeit) vergleichbar ein, überschätzten sie bei 5 (beispielsweise Fatigue) und unterschätzten sie bei 12 (unter anderem Verstopfung; Tab. 1). In Abb. 1 ist die durchschnittlich wahrgenommene Symptombelastung in absteigender Reihenfolge aus nephrologischer Sicht gezeigt, getrennt nach somatischen und psychischen Symptomen.

**Tab. 1:** Vergleich der Symptomhäufigkeiten und -belastungen

Merkmal	Nephrologinnen und Nephrologen		Patientinnen und Patienten		Vergleich
*n*	108		222		
	Häufigkeit	Symptombelastung	Häufigkeit	Symptombelastung	
	*n* (%)	M (SD)	*n* (%)	M (SD)	OR^a^/*t*^b^
*Somatische Symptome CKD-SBI*					
Verstopfung	105 (97,2 %)	3,22 (2,00)	40 (18,0 %)	4,75 (2,61)	**OR: 159,25; ***p* < 0,001; 95 %-KI [48,08; 527,45] ***t*(57,39)=−3,36; *p*=0,001**
Übelkeit	99 (91,7 %)	3,30 (2,02)	49 (22,1 %)	3,94 (2,52)	**OR: 38,84; ***p* < 0,001; 95 %-KI [18,30; 82,42] *t*(146) = −1,66; *p* = 0,098
Erbrechen	79 (73,1 %)	2,38 (1,92)	17 (7,7 %)	5,47 (3,22)	**OR: 32,85; ***p* < 0,001; 95 %-KI [17,11; 63,09] ***t*(18,52)=−3,81; *p*=0,001**
Durchfall	97 (89,8 %)	2,42 (1,76)	71 (32,0 %)	4,10 (3,02)	**OR: 18,75; ***p* < 0,001; 95 %-KI [9,46; 37,18] ***t*(104,21)=−4,20; *p*<0,001**
Verminderter Appetit	103 (95,4 %)	4,67 (2,08)	30 (13,5 %)	2,87 (2,00)	**OR: 131,84; ***p* < 0,001; 95 %-KI [49,65; 350,07] ***t*(131)=4,21; *p*<0,001**
Muskelkrämpfe	104 (96,3 %)	4,08 (1,96)	79 (35,6 %)	4,73 (2,73)	**OR: 47,06; ***p* < 0,001; 95 %-KI [16,71; 132,59] ***t*(135,21)=−1,80; *p*=0,074**
Schwellungen in den Beinen	108 (100 %)	5,28 (2,07)	76 (34,2 %)	3,91 (2,99)	**Χ**^**2**^**=127,39, *p*<0,001**^c^ ***t*(124,47)=3,46; *p*<0,001**
Kurzatmigkeit	107 (99,1 %)	4,74 (2,19)	87 (39,2 %)	4,77 (2,80)	**OR: 166,03; ***p* < 0,001; 95 %-KI [22,75; 1211,54] *t*(160,77) = −0,10; *p* = 0,924
Benommenheit oder Schwindel	102 (94,4 %)	3,52 (2,07)	66 (29,7 %)	3,68 (2,44)	**OR: 40,18; ***p* < 0,001; 95 %-KI [16,80; 96,12] *t*(166) = −0,46; *p* = 0,643
Unruhe in den Beinen oder Schwierigkeiten, die Beine still zu halten	104 (96,3 %)	3,90 (2,08)	33 (14,9 %)	3,88 (3,08)	**OR: 148,91; ***p* < 0,001; 95 %-KI [51,34; 431,92] *t*(41,64) = 0,04; *p* = 0,97
Taubheit oder Kribbeln in den Füßen	104 (96,3 %)	3,61 (2,03)	66 (29,7 %)	4,85 (3,09)	**OR: 61,46; ***p* < 0,001; 95 %-KI [21,74; 173,75] ***t*(100,78)=−2,88; *p*=0,005**
Müdigkeit oder Energielosigkeit/Fatigue	107 (99,1 %)	6,30 (2,06)	136 (61,3 %)	5,24 (2,64)	**OR: 67,66; ***p* < 0,001; 95 %-KI [9,27; 493,76] ***t*(241,00)=3,52; *p*<0,001**
Husten	91 (84,3 %)	2,39 (1,57)	78 (35,1 %)	3,74 (2,55)	**OR: 9,88; ***p* < 0,001; 95 %-KI [5,50; 17,77] ***t*(123,99)=−4,06; *p*<0,001**
Trockener Mund	98 (90,7 %)	2,82 (1,81)	58 (26,1 %)	4,16 (2,95)	**OR: 27,71; ***p* < 0,001; 95 %-KI [13,54; 56,72] ***t*(82,63)=−3,11; *p*=0,003**
Knochen- oder Gelenkschmerzen	103 (95,4 %)	3,70 (2,06)	95 (42,8 %)	5,37 (2,92)	**OR: 27,54; ***p* < 0,001; 95 %-KI [10,80; 70,22] ***t*(167,68)=−4,61; *p*<0,001**
Schmerzen im Brustkorb	97 (89,8 %)	2,34 (1,76)	26 (11,7 %)	4,69 (3,04)	**OR: 66,48; ***p* < 0,001; 95 %-KI [31,53; 140,14] ***t*(29,62)=−3,78; *p*<0,001**
Kopfschmerzen	100 (92,6 %)	3,22 (1,94)	62 (27,9 %)	4,08 (2,27)	**OR: 32,26; ***p* < 0,001; 95 %-KI [14,82; 70,20] ***t*(160)=−2,56; *p*=0,011**
Muskelschmerzen	100 (92,6 %)	3,30 (1,82)	58 (26,1 %)	4,91 (2,56)	**OR: 35,35; ***p* < 0,001; 95 %-KI [16,20; 77,10] ***t*(90,89)=−4,22; *p*<0,001**
Konzentrationsprobleme	106 (98,1 %)	4,95 (2,14)	69 (31,1 %)	4,58 (2,61)	**OR: 117,53; ***p* < 0,001; 95 %-KI [28,19; 489,86] *t*(173) = 1,02; *p* = 0,310
Trockene Haut	105 (97,2 %)	4,66 (2,16)	102 (45,9 %)	4,11 (2,90)	**OR: 41,18; ***p* < 0,001; 95 %-KI [12,68; 133,68] *t*(186,45) = 1,54; *p* = 0,125
Juckreiz	104 (96,3 %)	4,30 (2,22)	70 (31,5 %)	4,31 (2,87)	**OR: 56,46; ***p* < 0,001; 95 %-KI [20,00; 159,41] *t*(122,20) = −0,02; *p* = 0,983
Probleme beim Einschlafen	107 (99,1 %)	5,18 (2,22)	61 (27,5 %)	5,16 (3,00)	**OR: 282,41; ***p* < 0,001; 95 %-KI [38,56; 2068,11] *t*(97,95) = 0,04; *p* = 0,968
Probleme beim Durchschlafen	107 (99,1 %)	5,18 (2,22)	105 (47,3 %)	5,18 (2,89)	**OR: 119,23; ***p* < 0,001; 95 %-KI [16,35; 869,30] *t*(195,15) = 0,00; *p* = 0,998
Abnahme der Libido	106 (98,1 %)	4,66 (2,19)	54 (24,3 %)	4,56 (3,42)	**OR: 164,89; ***p* < 0,001; 95 %-KI [39,38; 690,49] *t*(75,86) = 0,21; *p* = 0,832
Probleme der sexuellen Erregbarkeit	104 (96,3 %)	4,65 (2,20)	46 (20,7 %)	4,57 (3,23)	**OR: 99,48; ***p* < 0,001; 95 %-KI [34,81; 284,29] *t*(64,18) = 0,16; *p* = 0,876
Unterbrechung des Nachtschlafs durch Harndrang	108 (100 %)	5,38 (2,29)	165 (74,3 %)	4,12 (2,88)	**Χ**^**2**^**=33,52, *p*<0,001**^**c**^ ***t*(260,68)=4,00; *p*<0,001**
*Somatische Symptombelastung*					
Somatische Symptombelastung (CKD-SBI), M (SD; Range)	94,56 (37,16; 25,56–190,00)		36,39 (32,98; 0–191)		***t*(328)=14,14; *p*<0,001**
Anzahl somatische Symptome (CKD-SBI), M (SD; Range)	24,58 (3,21; 8–26)		8,10 (4,99; 0–24)		***t*(303,23)=36,17; *p*<0,001**
*Psychische Symptome CKD-SBI*					
Grübeln/Sorgen	107 (99,1 %)	5,37 (2,19)	90 (40,5 %)	4,60 (2,60)	**OR: 156,93; ***p* < 0,001; 95 %-KI [21,51; 1144,89] ***t*(195)=2,25; *p*=0,025**
Nervosität	102 (94,4 %)	3,88 (2,26)	43 (19,4 %)	4,49 (2,80)	**OR: 70,77; ***p* < 0,001; 95 %-KI [29,12; 172,00] *t*(66,30) = −1,27; *p* = 0,210
Reizbarkeit	101 (93,5 %)	3,73 (1,99)	46 (20,7 %)	4,70 (2,80)	**OR: 55,21; ***p* < 0,001; 95 %-KI [24,03; 126,85] ***t*(66,46)=−2,11; *p*=0,038**
Traurigkeit	105 (97,2 %)	4,07 (2,30)	59 (26,6 %)	4,44 (2,65)	**OR: 96,70; ***p* < 0,001; 95 %-KI [29,55; 316,46] *t*(162) = −0,93; *p* = 0,356
Ängstlichkeit	104 (96,3 %)	4,38 (2,26)	34 (15,3 %)	4,47 (2,76)	**OR: 143,77; ***p* < 0,001; 95 %-KI [49,64; 416,35] *t*(136) = −0,19; *p* = 0,849
Depression	105 (97,2 %)	4,22 (2,27)	27 (12,2 %)	4,85 (2,90)	**OR: 252,78; ***p* < 0,001; 95 %-KI [74,92; 852,93] *t*(130) = −1,21; *p* = 0,229
*Psychische Symptombelastung*					
Psychische Symptombelastung (CKD-SBI), M (SD; Range)	24,75 (12,05; 0–54,44)		5,72 (10,03; 0–59)		***t*(181,30)=14,20; *p*<0,001**
Anzahl Symptome (CKD-SBI), M (SD; Range)	5,78 (0,86; 0–6)		1,35 (1,74; 0–6)		***t*(327,91)=31,03; *p*<0,001**
*Gesamtsymptombelastung (somatische und psychische Symptome)*					
Gesamtsymptombelastung (CKD-SBI), M (SD; Range)	119,31 (47,35; 25,56–233,33)		42,55 (40,43; 0–240)		***t*(185,10)=14,48; *p*<0,001**
Anzahl Symptome (CKD-SBI), M (SD; Range)	30,36 (3,91; 8–32)		9,45 (6,10; 0–30)		***t*(304,05)=37,59; *p*<0,001**

**Fettdruck** kennzeichnet signifikante Werte

^a^Vergleich der Häufigkeiten

^b^Vergleich der Symptombelastung

^c^Chi-Quadrat-Test verwendet aufgrund 100 %iger Zustimmung der Nephrologinnen und Nephrologen

*CKD-SBI* Chronic Kidney Disease Symptom Burden Index, *KI* Konfidenzintervall, *M* Mittelwert, *OR* Odds Ratio, *SD* „standard deviation“ (Standardabweichung)

### Aussagen über Symptombelastung

**Abb. 2:**
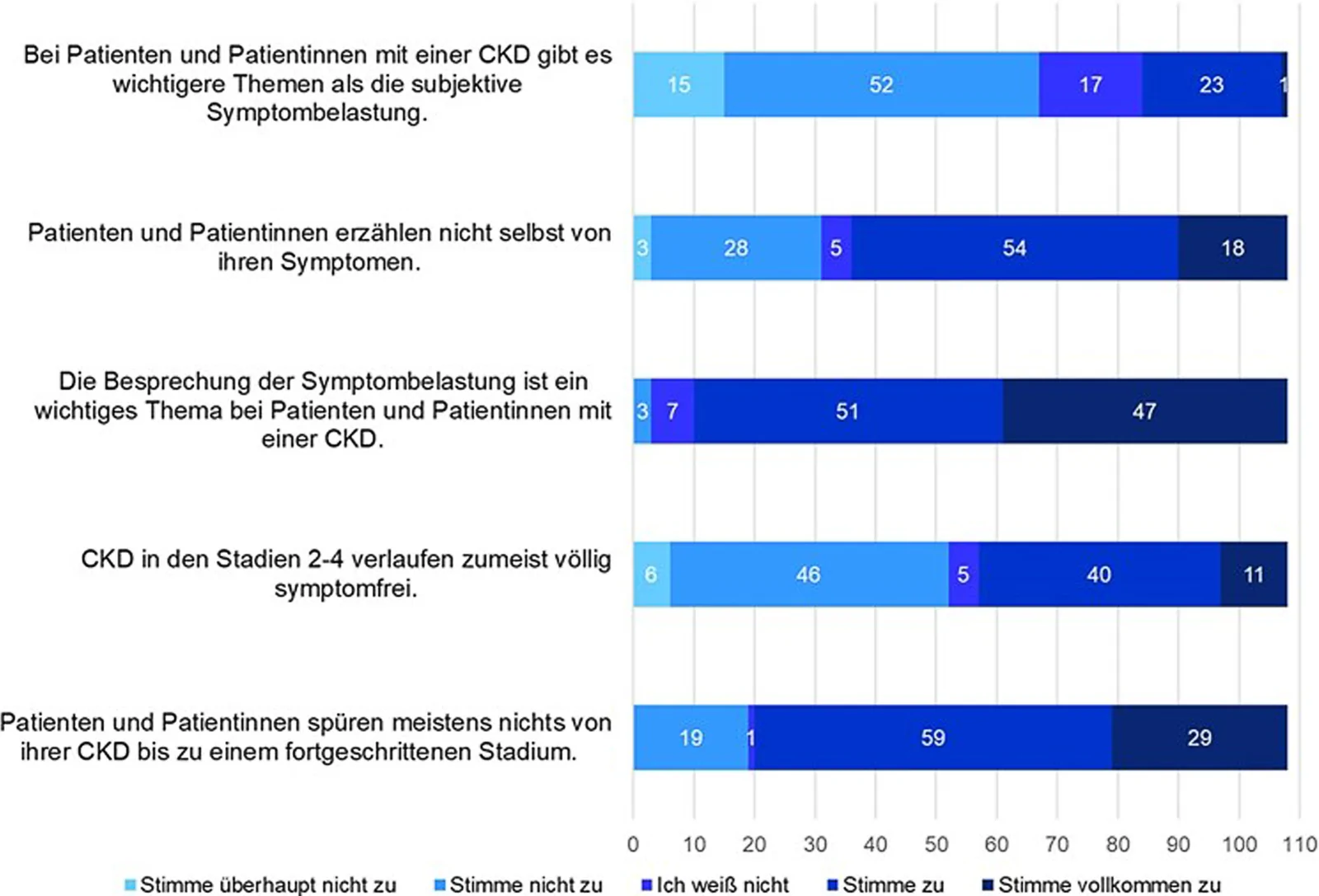
Zustimmung befragter Ärztinnen und Ärzte zu Aussagen über die Symptombelastung bei Personen mit CKD (Angabe von absoluten Zahlen). *CKD* „chronic kidney disease“ (chronische Nierenerkrankung)

Die Mehrheit der befragten Ärztinnen und Ärzte stimmte der Aussage zu, die Symptombelastung sowie deren Besprechung bei ND-CKD sei ein wichtiges Thema. Gleichzeitig wurde berichtet, dass Betroffene selten selbst von ihren Symptomen berichten. Bezüglich der Aussage, ob CKD in den Stadien 2–4 meist symptomfrei verlaufen, waren sich die befragten Ärztinnen und Ärzte uneinig (Abb. 2).

### Routinemäßige Symptomanamnese

Ärztinnen und Ärzte nannten in einem offenen Fragenformat typische Einstiegsfragen eines regulären Konsultationstermins bei Personen mit CKD. 52 (48,60 %) verwendeten *allgemeine Fragen zum Wohlbefinden *(„Wie geht es Ihnen?“), 42 (39,25 %) *Fragen zur Funktionalität *(„Wie ist Ihre Alltagsbelastung?“), 38 (35,51 %) *allgemeine symptombezogene Fragen* („Haben Sie Beschwerden?“) und 73 (68,22 %) *spezifische symptombezogene Fragen *(„Haben Sie Luftnot?“). Neun Fragen betrafen sonstige Themen. Tab. S2 bietet einen Gesamtüberblick über Fragen und Klassifizierung.

## Diskussion

Die Fachpersonenbefragung untersuchte die Einschätzung der Symptombelastung von Patientinnen und Patienten mit ND-CKD. Nephrologinnen und Nephrologen überschätzten sowohl die mit dem CKD-SBI erfasste nierenspezifische Gesamtsymptombelastung von Betroffenen mit CKD der SOMA.CK-Studie als auch die durchschnittliche Symptomanzahl signifikant. Die durchschnittliche eingeschätzte Symptombelastung von Einzelsymptomen wurde bei 15 Symptomen vergleichbar eingeschätzt, bei 12 unter- und bei 5 überschätzt. Im offenen Fragenformat der drei häufigsten Symptome stimmte die Einschätzung der Nephrologinnen und Nephrologen bezüglich der Häufigkeiten von Fatigue mit der von Betroffenen weitgehend überein. Demgegenüber wurden die von Betroffenen sehr häufig erlebten Symptome nächtlicher Harndrang und Schlafstörungen weniger wahrgenommen. Aus nephrologischer Sicht waren Ödeme und arterielle Hypertonie relevanter, wobei Letztere im Verständnis der Autoren kein subjektives Symptom, sondern eine Erkrankung darstellt. Odds Ratios zeigten, dass Nephrologinnen und Nephrologen die Häufigkeit jedes Symptoms des CKD-SBI signifikant höher einschätzten als Betroffene selbst. Diese Ergebnisse widersprechen bisheriger Forschung, die eher auf eine *Unterschätzung* der Symptombelastung durch nephrologische Fachpersonen hindeutet [4, 17, 26, 27]. Mit Blick auf die bisherigen Studien bleibt unklar, ob Symptome tatsächlich nicht erkannt oder nur unzureichend dokumentiert worden sind. Eine aktuelle Studie [28] zeigt, dass von Patient:innen berichtete Symptome bei CKD durch Nephrologinnen und Nephrologen nicht akkurat dokumentiert werden, was auf eine erhebliche Lücke in der Symptomkommunikation und einen Bedarf an standardisierten Erhebungsinstrumenten hinweist. Dies wird durch eine qualitative Studie unterstützt, in der Nephrologinnen und Nephrologen berichteten, unzureichend in der Anwendung von Patient-Reported Outcome Measures (PROM) sowie im Symptommanagement geschult zu sein [15], was die systematische Erfassung und Bewertung von Symptomen zusätzlich erschweren und zu abweichenden Einschätzungen beitragen kann.

Unklar bleibt, ob Symptome tatsächlich nicht erkannt oder nur unzureichend dokumentiert worden sind

Auch bei Erkrankungen wie myeloproliferativen Neoplasien [13], metastasierendem Brustkrebs [25] oder bei Patientinnen und Patienten mit Myelom [19] unterschätzten Ärztinnen und Ärzte die Symptomhäufigkeit bzw. -belastung [19]. Die Überschätzung des Vorhandenseins sowie der Gesamtsymptombelastung könnte hier durch die gezielte Befragung zu Symptomen und zur damit verbundenen Belastung sowie durch daraus resultierende sozial erwünschte Antworttendenzen entstanden sein. Die Rekrutierung der Nephrologinnen und Nephrologen für die Expertenbefragung fand unter anderem in Kooperationskliniken der SOMA.CK-Studie mit Fokus auf PSS bei CKD statt und könnte die Wahrnehmung und Sensibilität bezüglich der Symptombelastung bei Menschen mit CKD bei den Fachärztinnen und Fachärzten erhöht haben. Bei genauerer Betrachtung unserer Ergebnisse fällt auf, dass Nephrologinnen und Nephrologen die Symptombelastung von nur 5 Einzelsymptomen überschätzten (verminderter Appetit, Schwellungen in den Beinen, Fatigue, Nykturie und Grübeln/Sorgen), während sie die Symptombelastung von 12 unterschätzten und von 15 vergleichbar einschätzten. Andererseits zeigen unsere Ergebnisse, dass Befragte einige Symptome und die damit verbundene Belastung vergleichbar einschätzten oder sogar überschätzten. Jüngere Forschungsdaten im Bereich der Nierenerkrankung belegen hohe Symptomhäufigkeiten und eine hohe allgemeine Symptombelastung in allen CKD-Stadien, was zu zunehmendem Bewusstsein hierfür führt [21] und annehmen lässt, dass sich Nephrologinnen und Nephrologen der Symptombelastung bei CKD bewusst sind [7, 23].

Der CKD-SBI diente zur Erfassung nierenspezifischer Symptombelastung, im offenen Fragenformat nannten Nephrologinnen und Nephrologen hingegen häufig Erkrankungen bzw. mögliche Komorbiditäten wie Diabetes und Bluthochdruck [10]. Dies scheint auf ein abweichendes Verständnis subjektiver Symptombelastung bei den Autoren und Befragten hinzudeuten. Diese unterschiedlichen Symptomwahrnehmungen könnten darauf zurückzuführen sein, dass Nephrologinnen und Nephrologen jene Symptome stärker gewichten, die aus medizinischer Sicht auf ein Fortschreiten oder eine unzureichende Krankheitskontrolle hinweisen. Demgegenüber stehen für Betroffene eher Symptome im Vordergrund, die unmittelbare Einschränkungen im Alltag verursachen. In diesem Zusammenhang ist auch die eingeschränkte Vergleichbarkeit der beiden Gruppen zu berücksichtigen: Nephrologinnen und Nephrologen sollten Durchschnittswerte angeben, das heißt, welche Symptome ihre Patientinnen und Patienten insgesamt erleben, wohingegen Betroffene nach ihren persönlichen Symptomen befragt wurden. Dies könnte zusätzlich zur Überschätzung der Symptombelastung geführt haben. Zukünftige Forschung sollte daher nicht nur auf eine genaue Definition von Symptomen und Symptombelastung achten, sondern auch methodische Ansätze wählen, die eine bessere Vergleichbarkeit ermöglichen, wie beispielsweise die Befragung von Nephrologinnen und Nephrologen zu spezifischen Patientinnen und Patienten.

Verglichen mit einem systematischen metaanalytischen Review von Fletcher et al. [7] lagen berichtete Symptomhäufigkeiten der drei häufigsten Symptome, sofern berichtet, in einem ähnlichen Bereich (Vergleich Fatigue: Nephrologinnen und Nephrologen = 69,44 %, SOMA.CK = 61,30 %, Fletcher = 60–79 %; Schlafprobleme: SOMA.CK = 47,30 %, Fletcher 41–58 %). Beim Vergleich der Symptome aus dem CKD-SBI mit dem Review fällt auf, dass Nephrologinnen und Nephrologen auch hierbei die Symptomhäufigkeiten überschätzen, während die Häufigkeiten der SOMA.CK-Stichprobe größtenteils denen von Fletcher et al. ähneln (Beispiele: Schwellungen in den Beinen: Nephrologinnen und Nephrologen = 100 %, SOMA.CK = 34,2 %, Fletcher = 36–53 %; Übelkeit: Nephrologinnen und Nephrologen = 91,7 %, SOMA.CK = 22,1 %, Fletcher = 22–30 %).

Befragte stimmten der Aussage, die Thematisierung der Symptombelastung bei Menschen mit ND-CKD sei ein wichtiges Thema, mehrheitlich zu. Gleichzeitig berichteten die Befragten, dass Patientinnen und Patienten selten selbst von ihren Symptomen berichten, was möglicherweise darauf hindeutet, dass dieses Thema im klinischen Alltag unzureichend besprochen oder durch andere Themen wie die Behandlungsplanung oder Medikation überlagert wird. Dies stimmt mit früheren Forschungsdaten überein, gemäß denen Betroffene Symptome nur selektiv berichteten oder diese nicht eindeutig der Nierenerkrankung zuordnen konnten. Auch Schuldgefühle, die Zeit der Behandlerinnen und Behandler zu vergeuden, spielten hierbei eine Rolle [16].

In einer Implementierungsstudie wurde der routinemäßige Einsatz eines Screeningfragebogens zur Symptombelastung bei Personen mit Hämodialyse als umsetzbar postuliert und zeigte Verbesserungen des Symptombewusstseins sowohl auf nephrologischer als auch auf Betroffenenseite. Einschränkend ist jedoch zu berücksichtigen, dass Personen mit Hämodialysebehandlung im Vergleich zu Personen mit ND-CKD in der Regel mehrmals wöchentlich Dialyse erhalten und somit ein deutlich strukturierterer Zugang zu dieser Personengruppe besteht. Dennoch gab es kaum merkbare Verbesserungen bei der Symptombehandlung [6]. Zukünftig sollte überprüft werden, unter welchen Bedingungen ein verbessertes Bewusstsein für die Symptombelastung auch zu einem besseren Symptommanagement führt.

Ein routinemäßiges strukturiertes Symptomscreening könnte eine wertvolle Ergänzung sein

Die Auswertung routinemäßiger Symptomanamnesefragen zeigte, dass die Mehrheit der Nephrologinnen und Nephrologen allgemeine Fragen zum Wohlbefinden als Einstieg wählte („Wie geht es Ihnen?“), teils ergänzt durch allgemeine wie auch spezifische symptombezogene Fragen sowie Fragen zur Funktionalität. Ein routinemäßiges strukturiertes Symptomscreening könnte eine wertvolle Ergänzung im Sinne der Leitlinienempfehlungen zum Symptommanagement sein [10].

## Stärken und Limitationen

Diese Fachpersonenbefragung bietet wertvolle Einblicke in die Relevanz und Einschätzung der Symptombelastung bei ND-CKD in der klinischen Praxis und ermöglicht eine Einschätzung möglicher Diskrepanzen zwischen klinischer Wahrnehmung und von Betroffenen berichteter Symptomatik. Dennoch bestehen einige, insbesondere methodische, Limitationen: Die Vergleichbarkeit der beiden Gruppen ist eingeschränkt, da sich die Nephrologinnen und Nephrologen in ihren Einschätzungen auf Durchschnittswerte der von ihnen behandelten Individuen beziehen und nicht individuelle Teilnehmende der SOMA.CK-Studie bewerten. Der gewählte methodische Ansatz erlaubt entsprechend nur bedingt einen direkten Vergleich der Symptomhäufigkeiten bzw. -belastungen im Sinne einer *Über*- oder *Unterschätzung*. Vielmehr sollten die Ergebnisse als Ausdruck unterschiedlicher Perspektiven auf Symptomhäufigkeiten und -belastungen verstanden werden. Zudem wurde der CKD-SBI für die Fachpersonenbefragung adaptiert, sodass lediglich die Symptombelastung, nicht jedoch Vorhandensein, Ausprägung oder Häufigkeit des jeweiligen Symptoms erfragt wurde, was die Validität der direkten Gegenüberstellung beeinflussen könnte. Des Weiteren wurde nicht erfasst, ob das Fachpersonal in einer Praxis oder Klinik tätig ist, was die Wahrnehmung der Symptombelastung aufgrund des unterschiedlichen Patientenkollektivs beeinflussen könnte.

Unsere Rekrutierungsstrategie erlaubt keine Aussage über die Repräsentativität unserer fachärztlichen Stichprobe. Die breite Einladung über nephrologische Praxen und Fachgesellschaften ermöglicht keine genaue Schätzung der adressierten Grundgesamtheit, während durch die gezielte Rekrutierung über die Kooperationskliniken der SOMA.CK-Studie eine Verzerrung hinsichtlich einer höheren Sensibilität der teilnehmenden Nephrologinnen und Nephrologen für Symptombelastung nicht auszuschließen ist. Auch auf Betroffenenseite sind Selektionseffekte wie ein höheres Engagement oder stärker wahrgenommene Symptombelastung aufgrund der Studienteilnahme nicht auszuschließen.

## Ausblick

Die vorliegende sowie vergangene Studien betonen die Relevanz der Symptombelastung bei ND-CKD und das zunehmende Bewusstsein dafür [7, 23]. Diskrepanzen zwischen Behandelnden und Betroffenen in vergangenen Forschungsarbeiten und die Unterschätzung der Symptombelastung einiger Symptome in unserer Studie betonen die Notwendigkeit einer strukturierten Symptomerfassung im klinischen Alltag. KDIGO [10] und die Living Well With Kidney Disease and Effective Symptom Management Consensus Conference 2022 [18] empfehlen dies ebenfalls. Zukünftige Forschung sollte neben einer direkten Vergleichbarkeit der Aussagen von Betroffenen und Behandelnden auch eine routinemäßige nierenspezifische oder allgemeine Symptomerfassung beinhalten [5]. PROM wie der CKD-SBI eignen sich dafür [5, 14]. Allgemeine und spezifische symptombezogene Fragen sollten in regelmäßigen Arztkonsultationen zusätzlich gestellt werden. Für ein besseres Symptommanagement ist eine interdisziplinäre Zusammenarbeit unabdingbar, unter anderem durch Einbezug von Psychologinnen und Psychologen sowie anderen Fachdisziplinen [12, 21].

## Fazit für die Praxis

- Eine routinemäßige strukturierte Symptomerfassung bei nichtdialysepflichtiger chronischer Nierenerkrankung („non-dialysis chronic kidney disease“ [ND-CKD]) sollte Standard in der klinischen Praxis werden, beispielsweise durch Patient-Reported Outcome Measures (PROM) wie den Chronic Kidney Disease Symptom Burden Index (CKD-SBI).
- Bei erhöhter Symptombelastung sollte der Fokus auf der interdisziplinären Zusammenarbeit zwischen den Bereichen Nephrologie, Psychologie, Pflege, Ernährungsberatung und anderen Fachdisziplinen liegen – wie von Kidney Disease Improving Global Outcomes (KDIGO) empfohlen.
- Allgemeine Einstiegsfragen bei regulären Konsultationsterminen (beispielsweise „Wie geht es Ihnen?“) sollten um spezifische symptombezogene Fragen ergänzt werden.

## Supplementary Information


Tab. S1: Symptomhäufigkeiten aus nephrologischer SichtTab. S2: Antworten der befragten Nephrologinnen und Nephrologen zu ihrer routinemäßigen Symptomanamnese


## Data Availability

Alle dieser Arbeit zugrunde liegenden Daten sind in diesem Artikel enthalten.
