# Supplementary material for: Symptom burden in chronic kidney disease—nephrological assessment compared to patients’ perspectives (SOMA.CK study)
Source: Inn Med (Heidelb). 2026 Jul 22;67(9):1025–34. [Article in German] doi: 10.1007/s00108-026-02155-9 (PMC13529875; doi:10.1007/s00108-026-02155-9)
Supplement: Supplementary file 1 [file 108_2026_2155_MOESM1_ESM.docx]

# **Anhang**

Tab. S1: **Symptomhäufigkeiten aus nephrologischer Sicht**

| **Symptom** | **Anzahl (%)** |
| --- | --- |
| Fatigue | 75 (69,44%) |
| Ödeme | 42 (38,89%) |
| Hypertonie | 38 (35,19%) |
| Leistungsminderung | 32 (29,63%) |
| Juckreiz | 22 (20,37%) |
| Appetitlosigkeit | 21 (19,44%) |
| Dyspnoe | 19 (17,59%) |
| Anämie | 7 (6,48%) |
| Keine Symptome | 6 (5,56%) |
| Schmerzen | 6 (5,56%) |
| Schlafprobleme | 6 (5,56%) |
| Übelkeit | 6 (5,56%) |
| Antriebslosigkeit | 5 (4,63%) |
| Konzentrationsschwäche | 5 (4,63%) |
| Hyperkaliämie | 4 (3,70%) |
| Proteinurie | 3 (2,78%) |
| Schwindel | 3 (2,78%) |
| Depression | 3 (2,78%) |
| Adynamie | 2 (1,85%) |
| Hypervolämie | 2 (1,85%) |
| Hyperparathyreoidismus | 2 (1,85%) |
| Metabolische Azidose | 2 (1,85%) |
| Unwohlsein | 2 (1,85%) |
| Abneigung gegen Fleisch | 1 (0,93%) |
| Mangelernährung | 1 (0,93%) |
| Nykturie | 1 (0,93%) |
| Ängste | 1 (0,93%) |
| Uncharakteristische Myo-/Arthropathien | 1 (0,93%) |
| PTH Anstieg | 1 (0,93%) |
| UAW von Medikamenten | 1 (0,93%) |
| Laborbefund | 1 (0,93%) |
| Krämpfe | 1 (0,93%) |
| Hydrope Dekompensation | 1 (0,93%) |

Anmerkung: PTH = Parathormon, UAW = unerwünschte Arzneimittelwirkungen

Tab. S2: **Antworten der befragten Nephrologinnen und Nephrologen zu ihrer routinemäßigen Symptomanamnese**

| **ID** | **Allgemeine Fragen zum Wohlbefinden** | **Fragen zur Funktionalität** | **Allgemeine symptombezogene Fragen** | **Spezifische symptombezogene Fragen** | **Sonstiges** |
| --- | --- | --- | --- | --- | --- |
| **1** | Wie geht es Ihnen? | - | - | Haben Sie Luftnot bei Belastung? Gewicht, Trinkmenge, Ausscheidung? Haben Sie Beschwerden beim Wasserlassen? Wie ist der Blutdruck? | - |
| **2** | Mit Ihren eigenen Worten: was führt Sie in meine Sprechstunde? Fühlen Sie sich krank? | Gibt es in Ihrem Leben etwas, das nicht mehr so funktioniert wie früher? | Welche Beschwerden haben Sie in diesem Moment? Wie lange haben Sie diese Beschwerden schon? | - | - |
| **3** | - | - | - | Ab Stadium 4 Frage nach Müdigkeit, Abgeschlagenheit, Konzentrationsschwierigkeiten, Schlafstörung. Eigentlich bei allen Stadien Frage nach Ödemen und Blutdruck, falls Blutdruckmedikamente Frage nach Schwindel und Verträglichkeit. Immer Frage nach Luftnot. | - |
| **4** | - | - | - | Abfrage der typischen vegetativen Anamnese. Verdauung, Wasserlassen, Luftnot, Schlafstörungen, Leistung, Kopfschmerzen, Appetit, Gewichtsverhalten, Juckreiz | - |
| **5** | - | - | - | - | Abfragen |
| **6** | Allgemeine Einstiegsfrage ist immer offen gestaltet. Wie geht es Ihnen? Gibt es medizinisch etwas Neues, seitdem wir uns das letzte Mal gesehen haben? | - | - | Danach frage ich konkreter die Urämie-Symptome ab. Gewichtsverlust, Ödeme, Diarrhoe, Appetitverlust, Übelkeit, Konzentrationsschwäche, Pruritus, Müdigkeit, Luftnot, Leistungsverlust, etc. | - |
| **7** | - | - | - | Bei Erstvorstellung, je nach Fragestellung - gezielt - bei Mikrohämaturie (ob auch Episoden einer Makrohämaturie auftreten), bei Hypertonie, ob der Pat. Kopfschmerzen hat, Leistung-stabil? AP[Angina pectoris]-Symptomatik… bei WV [Wiedervorstellung] mit CKD- hat der Pat. seit letzter Vorstellung neue Beschwerden entwickelt, wenn ja welche? Im Stadium 5- Übelkeit, Appetit, Leistungsfähigkeit, Müdigkeit, Juckreiz… | - |
| **8** | - | Belastbarkeit im Alltag | - | Appetit | - |
| **9** | - | Belastbarkeit im Alltag, Treppensteigen, Radfahren bergauf möglich? | - | Blutdruckeigenmessung oder 24h-Messung erfolgt? Gewichtsverlauf, Ödeme an den Knöcheln? | - |
| **10** | Wie geht es Ihnen heute, hat sich seit dem letzten Termin etwas verändert? Ist Ihnen etwas aufgefallen? | Haben Sie Einschränkungen bei Ihrer Arbeit? Treiben Sie Sport? | - | die Fragen sind stark abhängig von den Komorbiditäten und den bisherigen Befunden/Symptomen sowie von den sozialen Faktoren;  haben Sie geschwollene Knöchel beobachtet? Wie sind Ihre Blutdruckwerte? – Was macht Ihr Appetit? – | - |
| **11** | - | - | - | Dyspnoe bei Belastung/Ruhe/Orthopnoe Ödeme/schwere/dicke Beine Appetenz/Gewichtschwankungen, Pruritus/Exanthem Arthritis Leistungsfähigkeit, Dysurie/Algurie/Urinpathologika | - |
| **12** | Einstieg (ganz offen): Wie geht es Ihnen? | - | - | Und dann gezieltes Fragen nach weiteren Symptomen | - |
| **13** | - | - | - | Essen Sie 3x/Tag? Haben Sie Juckreiz? Haben Sie Luftnot bei Belastung? | - |
| **14** | - | Leistungsfähigkeit, Gehstrecke | allgemeine Beschwerden | Frage nach Blutdruckwerten, Dyspnoe, Appetit, Übelkeit, Juckreiz | - |
| **15** | - | - | - | Fühlen Sie sich abgeschlagen/müde? Verspüren Sie Schmerzen (z.B. Kopf-/Bauchschmerzen o. andere), welche davor/früher nicht bestanden haben? Beklagen Sie Übelkeit o. neu aufgetretene Appetitlosigkeit? Ist Ihnen aufgefallen, dass Sie einen seltenen Harndrang haben, obwohl Sie die gleiche Menge Flüssigkeit zu sich nehmen? Oder lassen Sie weniger Urin(=Wasser) raus? Und wie ist es: haben Sie in der letzten Zeit das Gefühl gehabt, irgendwo am Körper Wasser (s.g. Ödeme) eingelagert zu haben (z.B. Fuß-/Unterschenkelbereich, Flanke oder gar im Gesichtsbereich)? Beklagen Sie neu aufgetretenen Juckreiz am Körper? | - |
| **16** | - | Fühlen Sie sich noch voll belastbar? | - | Sind Sie schneller erschöpft und müde? Hat Ihr Appetit nachgelassen? Können Sie sich noch gut konzentrieren? Wird die Luft knapp beim Treppensteigen? Haben Sie Juckreiz? | - |
| **17** | - | - | - | Gewichtsverlauf, Blutdruckselbstmessungen, Appetit, Atemnot, Juckreiz. | - |
| **18** | - | - | - | Haben sie Bauchschmerzen, Übelkeit? Sind Sie müde? | Was sagen Ihre Angehörigen? |
| **19** | - | - | Haben Sie Beschwerden? Gibt es gesundheitlich etwas Neues? | Wie sind RR und Diabetes eingestellt? | - |
| **20** | - | Haben Sie einen Leistungsknick bemerkt? Sind Sie weniger leistungsfähig als vor xx Jahren? | - | Können Sie gut durchschlafen? Sind Sie am Tag müde? | - |
| **21** | - | Wie ist Ihre Alltagsbelastung? | - | Haben Sie geschwollene Beine? Leiden Sie unter vermehrter Luftnot? Kopfschmerzen? Müssen Sie nachts auf Toilette? Wie häufig? | - |
| **22** | - | - | Was stört Sie im Moment am meisten? | Haben Sie geschwollene Beine? | - |
| **23** | - | - | - | Haben Sie Juckreiz, Ödeme, Appetitlosigkeit, (...), fühlen Sie sich abgeschlagen? | - |
| **24** | - | Wie ist Ihre körperliche Leistungsfähigkeit? Wie geht es Ihnen beim Treppensteigen? | - | Haben sie Kopfschmerzen, Herzbeschwerden? | - |
| **25** | - | - | Haben Sie seit der letzten Vorstellung Beschwerden gehabt? | - | - |
| **26** | - | Wie ist Ihre Belastbarkeit? Hat sich Ihre Belastbarkeit seit dem letzten Besuch verändert? | - | Haben Sie Wassereinlagerungen? Werden Ihre Beine abends dick? Können Sie nachts flach liegen? Wie oft müssen Sie nachts zum Wasserlassen auf Toilette?  Haben Sie Juckreiz? | - |
| **27** | Hat sich seit der letzten Kontrolle etwas geändert? | - | Neue Diagnosen, neue Medikamente, neue Beschwerden? | - | - |
| **28** | - | - | - | Ich frage die einzelnen Symptome ab, wenn sie nicht so benannt werden. Schlafen Sie gut? Schlafen Sie mehr als sonst? Wie ist Ihr Appetit? Schmeckt Ihnen das Essen und schmecken Sie was Sie essen? Was macht Ihre Haut? Fühlt sie sich trocken an? Müssen Sie sehr stark jucken? (Letzteres frage ich immer dann, wenn ich Kratzeffloreszenzen bei der körperlichen Untersuchung bemerke). | - |
| **29** | Ich frage direkt offen, dann konkret nach Symptomen wie Appetitlosigkeit, Übelkeit, Gewichtabnahme, Müdigkeit, Leistungsminderung, Juckreiz etc. Haben Sie bemerkt, dass Sie in letzter Zeit müder sind als sonst? etc. | - | - | Ich frage direkt offen, dann konkret nach Symptomen wie Appetitlosigkeit, Übelkeit, Gewichtabnahme, Müdigkeit, Leistungsminderung, Juckreiz etc. Haben Sie bemerkt, dass Sie in letzter Zeit müder sind als sonst? etc. | - |
| **30** | - | - | - | Klinische Untersuchung, Pat. gibt aktiv z.T. auch die Symptome wieder, beklagt diese, ansonsten direkte Nachfrage nach den Symptomen | Klinische Untersuchung, Pat. gibt aktiv z.T. auch die Symptome wieder, beklagt diese, ansonsten direkte Nachfrage nach den Symptomen |
| **31** | Wie ist das Allgemeinbefinden? | Körperliche Belastbarkeit? | - | Hautjucken oder Hautveränderungen? Luftnot unter Belastung Unterschenkelödeme? | - |
| **32** | - | Wie belastbar sind Sie, treiben Sie Sport? | - | Messen Sie selbst Ihren Blutdruck, wie sind die Werte? Sind die Beine abends manchmal dick? | - |
| **33** | - | - | - | Müdigkeit, eingeschränkte Leistungsfähigkeit, Kopfschmerzen, Hautjucken, Übelkeit, körperliche Schwäche | - |
| **34** | Offene Frage des Befindens, zusätzliche Erfragung spezifischer Symptome (z.B. Belastbarkeit, Dyspnoe, Pruritus, Nausea, Vomitus etc.) | Offene Frage des Befindens, zusätzliche Erfragung spezifischer Symptome (z.B. Belastbarkeit, Dyspnoe, Pruritus, Nausea, Vomitus etc.) | - | Offene Frage des Befindens, zusätzliche Erfragung spezifischer Symptome (z.B. Belastbarkeit, Dyspnoe, Pruritus, Nausea, Vomitus etc.) | - |
| **35** | Offene Frage: wie geht es Ihnen im Vergleich zum letzten Besuch in unserer Praxis? | - | - | Gezielte Fragen: wie hat sich Symptom X a) spontan oder b) durch die Behandlung mit Substanz X geändert? | Waren Sie in anderweitiger medizinischer Betreuung evtl. notfallmäßig? |
| **36** | - | Wie ist Ihre körperliche Belastbarkeit | Sind aktuell oder in den letzten Wochen Beschwerden aufgetreten? | - | - |
| **37** | - | - | - | Sind die Beine schlank? Wie ist der Blutdruck? Haben Sie Appetit? | - |
| **38** | - | Hat sich an Ihrer täglichen Lebensaktivität/-Qualität seit der letzten Untersuchung etwas verändert? | Sind seit der letzten Vorstellung neue Symptome/Beschwerden aufgetreten? | Hat sich Ihre Ernährung verändert? Haben Sie Luftnot in Ruhe/bei Belastung? | - |
| **39** | - | - | - | Sind Sie vermehrt müde? Haben Sie Luftnot? Haben Sie noch guten Appetit? Verspüren Sie Juckreiz? Können Sie sich gut konzentrieren? | - |
| **40** | Ansonsten wie bei allen: Wie fühlen Sie sich? | - | - | Standardfrage Stadium 4: Leiden Sie unter Müdigkeit, Appetitlosigkeit, Juckreiz, Übelkeit oder Durchfällen? | - |
| **41** | - | - | Subjektive Beschwerden? | - | - |
| **42** | - | Wie ist Ihre Belastbarkeit? Können Sie eine Etage Treppen steigen ohne Pause? | - | Viele Patienten mit Nierenerkrankung klagen über Müdigkeit. Wie ist das bei Ihnen? Wie ist das mit Juckreiz bei Ihnen? | - |
| **43** | - | - | Was belastet Sie aktuell am meisten? | - | - |
| **44** | Was fehlt Ihnen in der letzten Zeit, was haben Sie aktuell für Beschwerden? | - | Was fehlt Ihnen in der letzten Zeit, was haben Sie aktuell für Beschwerden? | - | - |
| **45** | Was führt Sie zu uns? | Hat Ihre Leistungsfähigkeit abgenommen? | - | Haben Sie in der letzten Zeit Schwellungen der Beine oder schäumenden Urin bemerkt? Messen Sie den Blutdruck? Zu welchen Zeiten messen Sie und wie waren die Werte? Ist Ihr Appetit gut? | - |
| **46** | - | - | Was für Beschwerden haben Sie? | Wie hoch ist Ihr Blutdruck? Was macht Ihr Appetit? Leiden Sie an Übelkeit? Haben Sie Luftnot? Haben Sie geschwollene Beine? | - |
| **47** | - | - | Was für Symptome haben Sie aktuell? | Haben sie Wassereinlagerungen? | - |
| **48** | Was gibt es Neues? | - | - | - | - |
| **49** | Was gibt es Neues? | - | - | - | - |
| **50** | Fühlen Sie sich wohl? Fehlt Ihnen was? | - | Was haben Sie an Beschwerden? | - | - |
| **51** | Was hat sich die letzte Zeit verändert, was ist neu? | - | - | Haben Sie Luftnot? Haben Sie an Gewicht zugenommen? Wie ist die Urinmenge? Wieviel Flüssigkeit trinken Sie am Tag? | - |
| **52** | Was hat sich geändert in den letzten Jahren? | Gibt es Aktivitäten, die Sie nur noch eingeschränkt durchführen können oder vielleicht gar nicht mehr? | Gibt es Symptome oder Auffälligkeiten, die Sie selber bemerkt haben, nach denen ich nicht explizit gefragt habe? | - | Fallen Personen in Ihrem Umfeld Dinge auf, auf die Sie angesprochen werden? |
| **53** | Was hat sich seit xx verändert? | - | - | Explizites Fragen: Wie gut ist der Nachtschlaf? Besteht xy? | - |
| **54** | - | Wie viele Treppenstufen kommen Sie ohne Probleme hoch? Wie geht Ihnen die Hausarbeit von der Hand? | - | Was macht der Appetit? Haben Sie Übelkeit oder Erbrechen? | - |
| **55** | - | - | Was merken Sie von Ihrer Nierenkrankheit? Welche Beschwerden haben Sie? Hat sich etwas an Ihrem Befinden verschlechtert? | Gezielte Symptomabfrage | - |
| **56** | - | Was schränkt Sie im Alltag ein? | - | - | - |
| **57** | - | - | Was stört Sie aktuell am meisten? | Haben Sie…? | - |
| **58** | - | Wie schätzen Sie Ihre Leistungsfähigkeit ein? | Welche Beschwerden beeinträchtigen Sie aktuell am meisten? | Haben Sie Veränderungen der Urinausscheidung bemerkt? Wie klappt es mit dem Essen? Ist der Appetit auf Essen gleichgeblieben? | - |
| **59** | - | - | Welche Beschwerden belasten Sie am meisten, die Sie früher nicht hatten? | Gezielte Abfrage von Symptomen Ja/ Nein (körperlich und psychisch) | - |
| **60** | - | Fühlen Sie sich aktuell im Alltag gut belastbar? | Welche Beschwerden haben Sie aktuell? | - | Gibt es etwas, was Sie mir gerne mitteilen wollen? |
| **61** | Hat sich an Ihrem Befinden etwas geändert? Wie fühlen Sie sich? | - | Welche Beschwerden haben Sie zurzeit? | - | - |
| **62** | - | - | Welche Beschwerden haben Sie? | - | - |
| **63** | - | - | Welche Beschwerden haben Sie? Was ist aktuell Ihr größtes Problem? | Oder konkreter: Haben Sie Luftnot? | - |
| **64** | - | - | Welche Beschwerden haben Sie aktuell? Haben Sie Beschwerden in der Nierengegend? | Haben Sie Auffälligkeiten beim Wasserlassen/Urin bemerkt? Haben Sie Wassereinlagerungen am Körper bemerkt? | - |
| **65** | Welche Dinge belasten Sie gerade/Wie geht es Ihnen? | Wie leistungsfähig sind Sie? Können Sie arbeiten gehen? Können Sie Ihre Freizeit aktiv gestalten? | Fällt Ihnen noch etwas ein, was Sie stört? Ist noch irgendetwas anders als früher? | Leiden Sie unter Erschöpfung/Müdigkeit? Haben Sie das Gefühl, sich schlechter konzentrieren zu können? Leiden Sie unter Juckreiz? Haben Sie Appetit? Wie viel essen Sie? Müssen Sie sich zum Essen zwingen? Haben Sie Durchfall, Übelkeit oder Erbrechen? Müssen Sie vermehrt schlafen? Haben Sie Wassereinlagerungen? Verändert sich Ihr Gewicht (mehr oder weniger)? Haben Sie Luftnot? | Haben Sie weitere Erkrankungen? |
| **66** | Haben Sie Juckreiz, Übelkeit, Wasser in den Beinen, Luftnot, Durchfall, Brennen beim Wasserlassen, fühlen Sie sich anders als sonst? | - | Welche Symptome haben sie aktuell? (Patientenantwort) | Folgend noch präziser: Haben Sie Juckreiz, Übelkeit, Wasser in den Beinen, Luftnot, Durchfall, Brennen beim Wasserlassen, fühlen Sie sich anders als sonst? | - |
| **67** | - | - | Welche Symptome haben Sie aktuell? Welche Symptome haben Sie vielleicht aktuell nicht, aber hin und wieder? Welche Beschwerden stehen für Sie im Vordergrund? | - | - |
| **68** | Welche Veränderungen haben Sie in den letzten Monaten wahrgenommen (Korrelation zur eGFR-Dynamik)? | - | - | Konkret nachfragen, je nach dem Stadium der Nierenfunktion. | - |
| **69** | - | - | Welche Veränderungen/Beeinträchtigungen haben Sie an Ihrem Körper in den letzten (Zeitintervall) bemerkt? Welche Beschwerden können Sie benennen? | Haben Sie (konkretes Symptom)? | - |
| **70** | Spüren Sie über das letzte Jahr / halbe Jahr eine Veränderung in Ihrem Wohlbefinden? | - | Welches Problem macht Ihnen im Moment am meisten zu schaffen? Welche Beschwerden haben Sie aktuell? | - | - |
| **71** | - | Wie bewältigen Sie Ihren Alltag? | - | Haben Sie Luftnot? | - |
| **72** | - | Können Sie Treppen gut steigen? | - | Wie fallen Ihre eigenen RR-Messungen aus? Haben Sie geschwollene Beine, vielleicht häufiger abends als morgens? Kommen Sie stets gut ohne Probleme in Ihre Schuhe? Hinterlassen die Socken Einschnitte? Haben Sie ungewollt an Körpergewicht zugenommen? Haben Sie Luftnot? Haben Sie bei der Alltagsbelastung Luftnot oder leiden Sie da an knapper Luft? | Können Sie flach liegen oder brauchen Sie viele Kissen? |
| **73** | Wie fühlen Sie sich aktuell? | Haben Sie Veränderungen in der körperlichen Belastbarkeit bemerkt? | - | Haben sie Luftnot? | - |
| **74** | Wie fühlen Sie sich aktuell? | - | Welche Beschwerden haben Sie? | - | - |
| **75** | Wie geht es Ihnen? | - | Gibt es irgendwelche Beschwerden? | - | - |
| **76** | Wie geht es Ihnen? | Können Sie Ihre täglichen Verrichtungen erledigen? | - | Haben Sie Luftnot beim Treppensteigen? | - |
| **77** | Wie geht es Ihnen? | Wie ist Ihre Belastbarkeit im Alltag, auf gerader Strecke, beim Treppensteigen? | - | Haben Sie geschwollene Beine? | - |
| **78** | Wie geht es Ihnen allgemein? | Können Sie Sport machen, sind sie weiter aktiv / gehfähig / in der Lage Treppen zu steigen? | - | Haben Sie Wassereinlagerungen? Bekommen Sie schnell Luftnot? | - |
| **79** | Wie geht es Ihnen heute? | - | - | Hatten Sie in den letzten Wochen eines der folgenden Symptome ... | - |
| **80** | Wie geht es Ihnen heute? | In welchen Bereichen waren Sie in den letzten 2 Wochen eingeschränkt? | - | - | - |
| **81** | Wie geht es Ihnen im Alltag? | Haben Sie genügend Energie, die täglichen Verrichtungen zu erledigen? | - | Blutdruckverhalten? Juckt die Haut? Appetit und Gewichtsverslauf? | Begleitung (z.B. Ehepartner) werden auch von mir befragt zum Allgemeinzustand der Patienten. |
| **82** | Wie geht es Ihnen zurzeit, was hat sich seit unserer letzten Visite geändert? | - | - | - | - |
| **83** | Wie geht es Ihnen, wie ist Ihre Belastbarkeit? | Wie geht es Ihnen, wie ist Ihre Belastbarkeit? | - | - | - |
| **84** | Wie geht es Ihnen? | - | - | - | - |
| **85** | Wie geht es Ihnen? | - | - | - | - |
| **86** | Wie geht es Ihnen? Was stört Sie derzeit am meisten? Was hat sich seit unserer letzten Besprechung verändert? | - | - | - | - |
| **87** | Wie geht es Ihnen? | Wie ist Ihre Belastbarkeit? | - | Wie ist Ihr Blutdruck? Haben Sie aktuell Wassereinlagerungen? | - |
| **88** | Wie geht es Ihnen? | - | Gibt es akute Probleme? | - | - |
| **89** | Wie geht es Ihnen? | Wie ist Ihre körperliche Belastbarkeit? | Haben Sie Beschwerden? | Gibt es Auffälligkeiten am Urin? | - |
| **90** | Wie geht es Ihnen? | Fühlen Sie sich leistungsfähig? Ist die Gehstrecke eingeschränkt? | - | Haben Sie Schmerzen? Wenn ja, sind diese belastungsabhängig? Treten Brustschmerzen auf? Treten Kopfschmerzen auf? Wenn ja, wie ist die Blutdruckeinstellung? Haben Sie Atemnot? Haben Sie Schlafstörungen? Wie ist die Stimmung, fühlen Sie sich niedergeschlagen? | - |
| **91** | Wie geht es Ihnen? | - | - | Haben Sie Übelkeit, Juckreiz oder ähnliches? | - |
| **92** | Wie geht es Ihnen? | - | - | Haben Sie Wasser in den Beinen? Haben Sie Beschwerden beim Wasser lassen? | - |
| **93** | Wie geht es Ihnen? Hat sich an Ihrem Zustand/Ihren Beschwerden etwas geändert? | - | Hat sich an Ihrem Zustand/Ihren Beschwerden etwas geändert? | - | - |
| **94** | Wie geht es Ihnen? | Sind Sie gut belastbar? Fühlen Sie sich fit? | - | - | - |
| **95** | Wie geht es Ihnen? So, wie es Ihnen immer schon ging, wenn es Ihnen gut ging? | Können Sie Ihren Alltag bewältigen? | - | Wie ist der Appetit? | - |
| **96** | Wie geht es Ihnen? | - | Welche Beschwerden haben Sie? | - | - |
| **97** | Wie geht es Ihnen? | - | - | Wie ist ihr Blutdruck? | - |
| **98** | Wie geht es/fühlen Sie sich? Wie ging es die letzten Monate? Gibt es Neuigkeiten? Allgemein: offene Fragen. | - | - | - | - |
| **99** | Wie geht ist Ihnen heute? | Wie sind Sie belastbar? | Haben Sie irgendwelche Beschwerden? | Haben Sie dicke Beine? Haben Sie Atemnot. Haben Sie Juckreiz? | - |
| **100** | - | - | - | Wie hoch ist Ihr Blutdruck? Haben Sie Ödeme? Leiden Sie an Atemnot? | - |
| **101** | - | Fühlen Sie sich belastbar? | - | Wie ist Ihr Appetit? Haben Sie Luftnot unter Belastung? Wasser in den Beinen? Schlafen Sie gut? | - |
| **102** | - | Wie ist Ihre Leistungsfähigkeit? | Gibt es neue Probleme? | - | - |
| **103** | Gab es seit der letzten Kontrolle Ereignisse, die Ihr Gesundheitsgefühl beeinträchtigt haben? | Wie kommen Sie im Alltag zurecht? Haben Sie körperliche Veränderungen bei den täglichen Verrichtungen festgestellt? | - | - | - |
| **104** | - | - | - | Wieviel trinken Sie? Wie oft müssen Sie Urin lassen? Müssen Sie auch nachts aufstehen? Schäumt sich Urin? Haben Sie geschwollene Beine oder Luftnot? Haben Sie Übelkeit/Erbrechen/Durchfall? | - |
| **105** | - | - | - | Wieviel wiegen Sie jetzt? Besteht Müdigkeit oder Juckreiz? | - |
| **106** | - | Wie viele Stufen/ Stockwerke können Sie ohne Pause steigen, bevor Sie Luftnot bekommen? Wie ist Ihre Belastbarkeit im Vergleich zu Ihren ehemaligen Klassenkameraden? | - | - | - |
| **107**^[1]^ | - | - | Worunter leiden Sie gerade am meisten? | Welche Schmerzen haben Sie? Haben Sie Wassereinlagerungen? Juckreiz? Schwäche? Blutdruckentgleisungen? | - |

Anmerkung: Grau Hinterlegtes gehört in andere Kategorien, die Frage wurde jedoch zusammenhängend genannt. Text in eckigen Klammern wurde von den Autoren ergänzt

1. Eine Person hat diese Frage nicht beantwortet.
